# The Attachment Type, Relationship Characteristics, and Sexual Function of Women: Insights from a Cross-Sectional Analysis

**DOI:** 10.3390/ijerph22050794

**Published:** 2025-05-18

**Authors:** Ioulia Kokka, Paraskevi Sotiropoulou, Iraklis Mourikis

**Affiliations:** First Department of Psychiatry, School of Medicine, National and Kapodistrian University of Athens, Outpatient Specialty Clinic for Sexual Health, Eginition Hospital, Vasilissis Sofia’s Ave. 72-74, 11528 Athens, Greece

**Keywords:** sexual health, attachment type, female sexual function, relationship satisfaction, observational study

## Abstract

Background: Research has underscored that the attachment type could explain the association between sexual behavior and relational satisfaction. This study aimed to explore the relationship between attachment type, sexual function, and relationship characteristics of adult women. Methods: This cross-sectional study included an a priori calculated sample of 304 women, who completed the Female Sexual Function Index, the Relationship Assessment Scale, and the Experiences in Close Relationships—Revised Scale. Results: Women with anxious attachment types reported higher levels of sexual arousal, orgasm, and satisfaction compared to other types, suggesting that emotional insecurity may intensify sexual intimacy within relationships. Overall, relational satisfaction was positively associated with various aspects of sexual function, particularly arousal and orgasmic function. Specifically, anxious attachment was associated with higher levels of sexual arousal, orgasm, and overall satisfaction, emphasizing the impact of relational dynamics on sexual well-being. Conclusions: The findings of this study highlight the significant role of attachment patterns and relational satisfaction in shaping women’s sexual function and experiences. The results suggest that therapeutic interventions aiming at the improvement of women’s sexual health should consider both attachment type and relational satisfaction as these factors are integral to the quality of sexual experience.

## 1. Introduction

Attachment theory [1,2,3,4] and its influence on sexuality has been extensively studied [5,6,7]. Previous research has suggested that attachment within romantic relationships is categorized into secure and insecure types [8]. Securely attached individuals form relationships based on trust and support, elements that promote relationship longevity and happiness [4]. On the contrary, insecurely attached individuals tend to either fear abandonment and, therefore, need others to reciprocate their feelings (anxious type) [9] or fear intimacy and closeness and distance themselves from others (avoidant type) [8].

Attachment type shapes the experience of sexual interaction [10]. Securely attached individuals engage in sexual activity in order to strengthen their bond with their partner [11]. Sexual experiences, marked by affection and exploratory behaviors, contribute to enhancing relationship quality and fostering long-term relational stability [10,12]. With respect to anxiously attached individuals, those seem to engage in sexual intercourse as a means to foster closeness and alleviate their insecurities [13] and fulfill their desire for emotional fusion [14,15] by prioritizing their partners’ sexual needs over their own [16]. Consequently, this behavioral pattern hinders the ability to experience physical sensations during intercourse [16], contributing to low sexual desire and other sexual difficulties [17,18]. Furthermore, anxiously attached individuals are more likely to engage in risky or unwanted sexual activities [19,20] as they seem to conflate sex with love, viewing sexual intimacy as a way to secure affection and relational closeness [15,21].

Avoidantly attached individuals perceive sexual connection and intimate affection differently [15,20,21]. They often engage in sexual activity for reasons unrelated to their relationship, like stress relief [15]. In addition, they are more likely to seek sexual experiences outside the relationship [19,22] and tend to masturbate more frequently than engage in sexual activity with their partners [22,23]. Sexual satisfaction is lower in avoidant and anxiously attached individuals [24,25,26,27]. Furthermore, these types of attachment are associated with difficulties in sexual arousal, lubrication, orgasm, and sexual pain in females [13,17,28,29] and erectile problems in males [23]. However, despite the rich body of international literature on attachment and sexual functioning, most existing studies have been conducted in Anglo-Saxon or Western European populations, limiting the generalizability of the findings to different cultural settings. Additionally, the majority of prior research has focused either on attachment or sexual satisfaction separately, rather than comprehensively investigating how attachment patterns, sexual function, and relationship characteristics interact.

Considering that the sexual system constitutes a fundamental component of most romantic relationships, it appears to play a pivotal role in fostering emotional intimacy and facilitating the maintenance and longevity of the partnership [20,30]. The attachment type could explain the association between sexual behavior and relationship satisfaction [31,32,33]. Individuals with an anxious attachment type tend to evaluate the quality of their relationship based on their sexual experiences; satisfying sexual encounters, which evoke feelings of love and connection, temporarily alleviate their fear of abandonment, thereby enhancing their perception of the overall relationship quality. Conversely, negative sexual experiences are interpreted as signs of their partner’s dissatisfaction, intensifying the anxiety about potential abandonment [2]. For anxiously attached individuals, specific sexual behaviors—such as increased frequency of activity—positively contribute to relationship satisfaction. Similarly, a negative connection has been found between avoidant attachment and relationship satisfaction, with more avoidant individuals reporting lower relationship satisfaction [32].

In the context of romantic relationships, individuals derive satisfaction from their partner’s affection, shared understanding, and emotional intimacy, all of which have been found to be positively associated with the benefits of sexual activity [34,35,36]. In addition, greater self-disclosure in long-term relationships is related to better sexual function [37]. Individuals with a stable partner report lower levels of anxiety than those engaging solely in casual sexual activity [38], indicating that non-committed sexual encounters may be linked with negative feelings, such as regret and guilt [39,40]. When sexual satisfaction is examined, men and women with a stable partner demonstrate greater satisfaction by their sexual life compared to those who engage in sexual activity outside of a committed relationship [38]. Despite these insights, relatively few studies have systematically examined the interplay between attachment type, specific domains of female sexual function (such as desire, arousal, orgasm, and satisfaction), and broader relationship characteristics like relationship type and duration, particularly in non-clinical samples. This multidimensional approach is essential for a more nuanced understanding of women’s sexual health.

Given the scarce evidence on sexual function from Greek samples [41,42], and the need for culturally sensitive research, the aim of the present study was to explore the relationship between attachment type, sexual function, and relationship characteristics of adult women from the general population.

## 2. Materials and Methods

### 2.1. Study Design

The present cross-sectional study was conducted between May of 2024 and November 2024. The study was held by the Outpatient Special Clinic of Sexual Health, First Psychiatric Department of the National and Kapodistrian University of Athens, Greece. It was consistent with the Declaration of Helsinki for human participants, and the research protocol was approved by the hospital’s Committee of Ethics (447/23-07-21). The study’s report was based on the STROBE Statement checklist for observational (cross-sectional) studies.

### 2.2. Participants

The study group was recruited from the community, using the snowball sampling technique. To mitigate biases regarding the sampling method, multiple different entry points were used. The sample size was determined using the G* Power software (version 3.1) [43]. A medium effect size (*ρ* = 0.3) was selected based on the prior literature and the expected strength of the relationships under investigation. The desired statistical power (1 − *β*) was set at 0.95 to minimize the risk of Type II errors. Based on these parameters, the minimum required sample size for the analyses was calculated to be 179 participants. Specific eligibility criteria were required for participation. Due to the premenopausal and menopausal impact on the female sexual function [44,45,46], women had to be 18 to 45 years old, be in an intimate relationship for at least the past 6 months, and be sexually active for at least the past four weeks. Due to the impact of psychiatric diagnoses and medication on sexual function [47], women with an active psychiatric diagnosis and relevant medication were excluded. Pregnant women were also excluded, as pregnancy may also affect the female sexual function [48]. Responses with missing values were not included in the analyses. Sexual orientation and the type of relationship did not constitute criteria of eligibility.

### 2.3. Measures

Participants completed a demographic characteristics’ form as well as a battery of self-report questionnaires regarding the variables under evaluation. All self-report instruments have been previously used and validated in the Greek language.

Demographic characteristics: participating women reported their basic demographic characteristics (age, sexual orientation, educational level, and relationship type and duration). Women also declared having an active psychiatric diagnosis and whether they were aware of being pregnant at the point of participation.

Female Sexual Function Index: Designed to evaluate the female sexual function, the FSFI consists of 19 items (e.g., “Over the past 4 weeks, how often did you feel sexual desire or interest?” and “Over the past 4 weeks, how would you rate your level (degree) of sexual desire or interest?” and six subscales (desire, arousal, lubrication, orgasm, satisfaction, and pain) [49,50]. Based on the Greek validation of the instrument, scores lower than 26 indicate increased risk of sexual dysfunction. Internal consistency (Cronbach’s α) of the instrument for the present study was 0.92.

Relationship Assessment Scale—RAS: The scale consists of seven items (e.g., “how well does your partner meet your needs?” and “how many problems are there in your relationship?”) and is designed to evaluate satisfaction from romantic, intimate relationships [51,52]. Responses are given on a 5-point Likert-like scale. The scale does not have cut-off scores, but higher total scores indicate greater relationship satisfaction. Internal consistency (Cronbach’s α) of the instrument for the present study was 0.73.

Experiences in Close Relationships—Revised: Designed to evaluate adult attachment based on the two-dimensional model (avoidance–anxiety) [53,54], it consists of 36 items (e.g., “I am afraid that I will lose my partner’s love” and “I prefer not to show a partner how I feel deep down”) referring to intimate relationships. It includes two subscales of 18 items, each assessing the two dimensions of adult attachment. Responses are given on a 7-point Likert-like scale. In alignment with the model proposed by Bartholomew and Horowitz [55], total scores of responses (high avoidance and anxiety, low avoidance and anxiety, low avoidance/high anxiety, and high avoidance/low anxiety) categorize the respondent under the (a) anxious, (b) avoidant, (c) fearful, and (d) secure attachment type. For the purposes of the study, the sample’s mean scores were used as cut-off scores for the two dimensions. Internal consistency (Cronbach’s α) of the instrument for the present study was 0.83.

### 2.4. Statistical Analyses

The descriptive statistics of demographic characteristics and the self-report measures are presented in terms of means and standard deviations, relative (%) and absolute (N) values, and medians and interquartile ranges for data that did not follow a normal distribution. The normality of data was checked with the Kolmogorov–Smirnov statistical test, graphical methods (histograms and Q–Q plots), and descriptive indices (skewness and kurtosis). Several variables showed notable deviations from normality, with skewness and/or kurtosis values exceeding recommended thresholds. Accordingly, non-parametric tests were used in the analysis. Correlations among continuous variables were checked with the Spearman’s *rho*. The correlation among categorical variables (attachment type and type of relationship) were examined with the Kruskal–Wallis *H* Test. Post hoc pairwise comparisons between types of attachment were performed with the Mann–Whitney *U* Test, using Bonferroni corrections (for 6 comparisons, *p* < 0.0083). The analyses were performed using the Statistical Package for Social Sciences (SPSS v29.0).

### 2.5. Ethical Considerations

Eligible candidates were thoroughly informed about the study’s purposes and were recruited only after they provided their consent. Participants were asked to provide a 6-digit/characters code in order to ensure anonymity and maintain the ability to identify responses in case of withdrawal. The collected data were registered in Excel worksheets and stored electronically. Access was available solely to the research team. No financial or other type of remuneration was provided for participating.

## 3. Results

### 3.1. Flow of Study Participation

Initially, 369 women participated in the study. Eight responses were excluded due to missing values. Among the 351 complete participations, 49 responses were excluded from analyses due to an active psychiatric diagnosis and eight because of a positive pregnancy. Finally, 304 responses fulfilled the eligibility criteria and were included in the analyses.

### 3.2. Sample’s Demographic Characteristics and Descriptive Statistics of Measurements

The mean age of the sample was 28.3 years, while the mean duration of their relationships in months was 50.4. Among the 304 participants, 83.6% were of heterosexual orientation (3.2% of homosexual and 11.3% of bisexual orientation), and 68.4% were in an exclusive monogamous relationship (16.4% were married, 10.2% in open monogamous relationships, and 4.9% in open polygamous relationships). In total, 92.4% were of higher education, and 55.6% were employees of the public or private sector. The descriptive statistics of the sample’s results on the self-report measures that were applied are being presented in Table 1.

### 3.3. Correlations Between Relationship Satisfaction, Relationship’s Duration, and Sexual Function

The Kolmogorov–Smirnov test indicated that the FSFI, RAS, and ECR–R significantly deviated from a normal distribution (*p* < 0.05). The relationship between continuous variables (relationship satisfaction, relationship’s duration, and sexual function) was tested with the Spearman’s *rho* coefficient. The results indicated that the duration of the relationship was significantly correlated in a positive and fairly weak fashion only with the FSFI’s subscale of desire (*rho* = 0.283), whereas relationship satisfaction was positively correlated in a weak to moderate way with the subscales of arousal (*rho* = 0.201, *p* < 0.01), lubrication (*rho* = 0.141, *p* = 0.014), orgasm (*rho* = 0.228, *p* < 0.01), satisfaction (*rho* = 0.378, *p* < 0.01), and the total score of the FSFI (*rho* = 0.276, *p* < 0.01). Detailed results of the correlations are presented in Table 2.

### 3.4. Differences in Sexual Function Across Attachment Types

The comparison between categorical variables (the attachment type and the type of relationship) with female sexual function was examined with the Kruskal–Wallis H Test, with Bonferroni corrections applied for multiple comparisons (α_adjusted_ = 0.0083).

The results revealed a significant difference across the four attachment types in the arousal subscale [*H* (3) = 13.28, *p* = 0.004] with Dismissive–Avoidant attachment demonstrating the lowest scores (mean rank = 134.63) and the orgasm subscale [*H* (3) = 22.57, *p* < 0.001] with Dismissive–Avoidant attachment showing lower subscale scores (mean rank = 123.56). Additionally, significant relationships were observed between the attachment type and satisfaction [*H* (3) = 31.54, *p* < 0.001] with Dismissive–Avoidant attachment demonstrating the lowest levels of satisfaction (mean rank = 125.83), and the total score of the FSFI [*H* (3) = 13.27, *p* = 0.004] with the same attachment type demonstrating the lowest total scores (mean rank = 134.73).

Post hoc analysis was conducted with the Mann–Whitney *U* Test to determine which pairs of groups differed significantly in the subscales of sexual function that arose from the Kruskal–Wallis *H* Test. Bonferroni corrections were applied to account for multiple comparisons to avoid type II errors and maintain the adjusted significance level (α_adjusted_ = 0.0083). The analysis revealed significant differences across the subscales of arousal, orgasm, satisfaction, and the total scores of the FSFI. A significant difference was observed between the anxious and avoidant attachment type regarding the arousal, with anxious participants demonstrating higher mean ranks (*p* < 0.0001). In the subscale of orgasm, significant differences were found between secure and avoidant attachment type, with securely attached individuals scoring higher (*p* = 0.002), and between anxious and avoidant type, with anxious individuals scoring higher (*p* < 0.0001). Regarding the subscale of satisfaction, significant differences were reported between secure and anxious attachment type; anxious individuals demonstrated higher mean ranks (*p* = 0.001). The results were similar for the pairs anxious and avoidant (*p* < 0.01) and anxious and fearful type (*p* = 0.002), with anxiously attached individuals consistently scoring higher. Regarding the total scores of the FSFI, a significant difference was observed between anxious and avoidant attachment type, with anxious individuals showing higher ranks (*p* < 0.0001). Detailed results of the test are presented in Table 3.

### 3.5. Differences in Sexual Function Based on Relationship Type

The results regarding the comparison of sexual function based on the type of relationship was also examined with the Kruskal–Wallis H Test, with Bonferroni corrections applied for multiple comparisons (α_adjusted_ = 0.0083). The results revealed a significant difference across the four types of relationship only for the subscale of desire [*H* (3) = 13.72, *p* = 0.003], with those being in an open polygamous relationship demonstrating the lowest scores (mean rank = 125.27).

Regarding the differences in sexual desire across the four relationship types, the Mann–Whitney *U* Test revealed that married participants reported significantly higher levels of desire compared to those in open monogamous relationships (*U* = 472.00, *Z* = −2.99, *p* = 0.003), as well as compared to those in exclusive monogamous relationships (*U* = 3678.50, *Z* = −3.259, *p* = 0.001). Detailed results of the Mann–Whitney *U* Test are presented in Table 4.

## 4. Discussion

Attachment patterns, formed early in life, significantly shape adult romantic and sexual relationships [1,3,8]. To the authors’ knowledge, this is the first study exploring the relationship between attachment type, sexual functioning, and relationship characteristics of Greek adult women. Key findings emerged and, in line with the initial hypothesis, attachment, and relationship type, relationship duration and satisfaction were associated with aspects of sexual health.

The primary aim of this study was to explore the relationship between attachment type and sexual function. Significant differences emerged between the four types and the subscales of arousal, satisfaction, orgasm, and the total score of the FSFI. In all dimensions except for lubrication, the avoidant attachment type group reported the lowest scores, indicating sexual difficulties almost globally. This finding is consistent with previous research, indicating that women of avoidant attachment tended to present with difficulties across all domains of sexual function [56]. Women of avoidant attachment type often experience difficulties being emotionally intimate and vulnerable [57]. Their tendency to detach emotionally [58] might diminish sexual desire, arousal, and overall satisfaction as they may struggle to find meaning in intimate experiences. Furthermore, negative beliefs of avoidantly attached individuals about dependence [59] may explain the decreased motivation for sexual and emotional engagement. Even though these results were probably expected, the between-attachment groups comparisons revealed a surprising finding; anxiously attached women scored higher on sexual satisfaction compared to securely attached individuals. This could be explained by the fact that the anxious-attachment-type group of this study scored high in sexual desire, lubrication, and orgasm, resulting in high sexual satisfaction. According to the literature, anxiously attached individuals tend to conflate sexual intimacy with emotional attachment and romantic love [15,20,21,60]. Consequently, they often interpret satisfying and rewarding sexual experiences as indicative of a high-quality and fulfilling romantic relationship. This pattern can also be interpreted through broader relational models such as the Investment Model [61] and the Social Exchange Theory [62], both of which suggest that individuals who perceive greater emotional investment and relational rewards report higher satisfaction, even when relational insecurity is present. Therefore, anxious individuals’ heightened focus on emotional closeness and investment might amplify their subjective satisfaction even in sexual contexts. While these models were not the primary theoretical framework of the present study, they offer complementary perspectives that enrich the understanding of the observed associations.

The secondary aim of the present study was to investigate the possible connection between relationship duration, type, and satisfaction and aspects of sexual function. Relationship duration was significantly associated with the subscale of desire of the FSFI, indicating that the longer the relationship, the greater the desire. This finding contradicts relevant research, which has shown that sexual desire diminishes as relationships mature [31,63,64]. Previous research suggests that during the early stages of a relationship, sexual desire is primarily driven by superficial partner characteristics, such as physical attractiveness and friendliness [65,66]. Nevertheless, over time, desire appears to be influenced by deeper qualities, such as sensitivity and responsiveness [67], which tend to emerge as the relationship develops. These findings align with Basson’s (2001) theoretical framework of the human sexual response cycle, which posits that emotional intimacy and affective connection play a crucial role in sustaining sexual desire [68]. Accordingly, it is possible that women experience an increase in sexual desire as the relationship progresses as it is during later stages that they derive greater satisfaction from their partner’s emotional attunement and bonding, leading to reduced anxiety and enhanced sexual fulfillment. Another plausible explanation for these contradictory results could be societal expectations. Women, in order to be considered fulfilled, are expected to be partnered [69] and “good, nice, and silent” to avoid the loss of the relationship [70]. Therefore, even women in long-term relationships who internalize these expectations might report high levels of desire. A similar interpretation is supported by longitudinal studies investigating relationship trajectories over time. For instance, research has shown that emotional investment and perceived relational commitment, key constructs of both the Investment Model and Social Exchange Theory, predict higher relationship and sexual satisfaction longitudinally [71,72]. Thus, it is plausible that in long-term relationships where emotional investment remains high, women report sustained or even enhanced sexual desire.

This study, also, examined the relationship between aspects of sexual function and relationship satisfaction, with the results revealing a positive association between relationship satisfaction and the subscales of arousal, lubrication, orgasm, and satisfaction and the total score of the FSFI questionnaire. These findings came in line with relevant theory and research. Bason has suggested that, for women, emotional intimacy is believed to stimulate sexual desire and, simultaneously, act as a reward of sexual arousal and orgasm [73]. The research findings have, also, highlighted the relation between aspects of sexual function and relationship satisfaction. For example, lubrication and orgasm have continuously risen as critical components of relational well-being and satisfaction [29,74].

With respect to the connection between sexual and relationship satisfaction, the present study reported a positive association between the two variables, confirming theoretical perspectives and other research findings. The interpersonal exchange model of sexual satisfaction suggests that the perceived relationship quality affects sexual satisfaction [75], while relevant research has shown that sexual satisfaction is positively connected to relational satisfaction [76,77]. However, it must be noted that the present study could not detect a causal relationship, and other research evidence has noted that sexual satisfaction exerts an influence on relationship satisfaction, whereas the reverse does not hold true [78].

Regarding the type of relationship and its relation to sexual function, the comparison reported a significant difference only for the subscale of desire, with further analysis showing that those being married reported significantly higher levels compared to those in exclusive and open monogamous relationships. This finding appears as striking since a large portion of the literature has shown that being in a long-term relationship negatively affects women’s sense of differentiation from the partner and, consequently, the level of sexual desire [79]. However, a growing body of research has examined the relational factors that contribute to the maintenance of desire over time and suggests that high levels of emotional closeness and intimacy between partners is the key route to desire maintenance in long-term relationships [80,81].

The present study demonstrates specific strengths and limitations that need to be acknowledged. The a priori power calculation of the sample size allows the generalization of the findings in a trustworthy manner, while the adjustments for significance thresholds ensure that the observed effects are not merely due to chance. Furthermore, the integration of theoretical frameworks from attachment and sexual functioning fields enables a richer interpretation of the results, contributing to both clinical practice and research. Despite the strengths, this study bears certain limitations that should be addressed. Even though hormonal contraceptives have been shown to influence aspects of sexual function and relationship dynamics, findings in the literature remain inconsistent [82]. Therefore, hormonal contraception use was not recorded; as such, a criterion could have restricted the recruitment process. Nevertheless, future research should consider controlling for contraceptive use to further clarify its potential impact on sexual function and relational variables. Moreover, although attachment was categorized based on ECR–R scores to enhance clinical interpretability, it is acknowledged that a dimensional approach might provide a more nuanced understanding of attachment patterns [83]. The cross-sectional study design precludes causal inferences, highlighting the need for cohort studies to better capture the temporal dynamics between the attachment type, relationship satisfaction, and sexual function. Additionally, the study relied exclusively on self-report measures, which may introduce biases such as social desirability and recall bias [84]. Lastly, the study utilized a snowball sampling method, which, while effective in reaching a diverse community sample, may introduce selection bias [85] by overrepresenting individuals with similar sociodemographic characteristics.

Future research should further explore the mechanisms through which attachment patterns influence specific domains of female sexual function, particularly through the lens of relational models such as the Investment Model and the Interpersonal Exchange Model of Sexual Satisfaction. Longitudinal designs would be valuable to clarify the directionality and stability of these associations over time. Moreover, examining the moderating role of cultural norms and gender expectations could offer critical insights into the socio-cultural shaping of attachment and sexual health outcomes. Finally, adopting a dimensional rather than categorical approach to attachment could provide a more nuanced understanding of individual differences in sexual functioning.

## 5. Conclusions

This study highlighted the role of the attachment type and relational dynamics in shaping women’s sexual functioning and satisfaction. The findings demonstrated that not only sexual function separately but also relationship satisfaction are key components of positive sexual experiences for women. In particular, the detected associations suggest that emotional needs within intimate relationships significantly modulate sexual health outcomes.

These results emphasize the importance of integrating relational factors into clinical interventions for sexual dysfunction, moving beyond a purely biomedical or individual-centered model. Furthermore, they point to broader gender-based issues, such as the emotional effort women often invest in relationships and the societal pressures regarding intimacy and performance. Addressing these structural and relational factors is essential for promoting women’s sexual health and enhancing their overall relational well-being. Given the complex interplay between attachment patterns, relational satisfaction, and sexual function, future research should investigate how these factors interact over time and across different cultural contexts. Longitudinal studies would be particularly valuable in exploring how changes in relationship dynamics influence women’s sexual health outcomes. Furthermore, examining the role of gender-based social expectations and emotional labor within intimate relationships will enhance the understanding of the socio-cultural determinants of sexual well-being.

## Figures and Tables

**Table 1:** Descriptive statistics of the sample’s responses to the self-report measures (*n* = 304).

**Self-Report Measure**	**Median**	**IQR**
Relationship Satisfaction	25	23; 27
FSFI Desire	5	4; 6
FSFI Arousal	7	5; 10
FSFI Lubrication	12	12; 12
FSFI Orgasm	8	7; 9
FSFI Satisfaction	6	4; 8
FSFI Pain	15	12; 15
FSFI total	51	46; 58
**Attachment Type (Based on ECR–R)**	***n***	**%**
Secure	44	14.4
Anxious	97	31.8
Dismissive–Avoidant	113	37.2
Fearful–Avoidant	50	16.4

Abbreviations: IQR = interquartile range; ECR–R = Experiences in Close Relationships—Revised; FSFI = Female Sexual Function Index.

**Table 2:** Spearman’s rho coefficient between relationship duration and satisfaction and FSFI.

	Spearman’s Coefficient	Desire	Arousal	Lubrication	Orgasm	Satisfaction	Pain	FSFI Total
Relationship duration	rho	0.28	−0.001	−0.07	−0.10	0.02	0.02	0.05
	*p*-value	<0.01	0.985	0.184	0.066	0.641	0.718	0.318
Relationship satisfaction	rho	0.10	0.20	0.14	0.22	0.37	0.004	0.27
	*p*-value	0.064	<0.01	0.014	<0.01	<0.01	0.948	<0.01

**Table 3:** Comparison of attachment styles on arousal, orgasm, satisfaction, and FSFI total scores using Mann–Whitney *U* Test.

	Attachment Type	Mean Rank	Sum of Ranks	Mann–Whitney *U* Test	Z	*p*-Value
**Arousal**	Secure vs. Anxious	60.17	2647.50	1657.50	−2.13	0.033
		75.91	7363.50			
	Secure vs. Avoidant	80.76	3553.50	2408.50	−0.30	0.759
		78.31	8849.50			
	Secure vs. Fearful	45.32	1994.00	1004.00	−0.73	0.463
		49.42	2471.00			
	Anxious vs. Avoidant	121.66	11,801.50	3912.50	−3.59	<0.01 *
		91.62	10,353.50			
	Anxious vs. Fearful	77.69	7535.50	2067.50	−1.46	0.142
		66.85	3342.50			
	Avoidant vs. Fearful	78.69	8892.50	2451.50	−1.35	0.175
		89.47	4473.50			
**Orgasm**	Secure vs. Anxious	70.27	3092.00	2102.00	−0.14	0.885
		71.33	6919.00			
	Secure vs. Avoidant	96.01	4224.50	1737.50	−3.03	0.002 *
		72.38	8178.50			
	Secure vs. Fearful	50.64	2228.00	962.00	−1.06	0.286
		44.74	2237.00			
	Anxious vs. Avoidant	124.83	12,108.50	3605.50	−4.40	<0.01 *
		88.91	10,046.50			
	Anxious vs. Fearful	77.86	7552.50	2050.50	−1.56	0.118
		66.51	3325.50			
	Avoidant vs. Fearful	76.27	8619.00	2178.00	−2.46	0.014
		94.94	4747.00			
**Satisfaction**	Secure vs. Anxious	54.41	2394.00	1404.00	−3.26	0.001 *
		78.53	7617.00			
	Secure vs. Avoidant	84.92	3736.50	2225.50	−1.03	0.302
		76.69	8666.50			
	Secure vs. Fearful	46.53	2047.50	1057.50	−0.32	0.745
		48.35	2417.50			
	Anxious vs. Avoidant	129.75	12,585.50	3128.50	−5.40	<0.01 *
		84.69	9569.50			
	Anxious vs. Fearful	81.74	7928.50	1674.50	−3.08	0.002 *
		58.99	2949.50			
	Avoidant vs. Fearful	78.45	8865.00	2424.00	−1.46	0.143
		90.02	4501.00			
**FSFI total score**	Secure vs. Anxious	61.17	2691.50	1701.50	−1.92	0.054
		75.46	7319.50			
	Secure vs. Avoidant	83.49	3673.50	2288.50	−0.77	0.440
		77.25	8729.50			
	Secure vs. Fearful	47.51	2090.50	1099.50	−0.004	0.997
		47.49	2374.50			
	Anxious vs. Avoidant	121.88	11,822.50	3891.50	−3.62	<0.01 *
		91.44	10,332.50			
	Anxious vs. Fearful	78.88	7651.50	1951.50	−1.93	0.053
		64.53	3226.50			
	Avoidant vs. Fearful	80.04	9045.00	2424.00	−1.46	0.143
		86.42	4321.00			

* Statistical significance set at *p* < 0.0083 after Bonferroni corrections.

**Table 4:** Comparison of relationship types on sexual desire using Mann–Whitney *U* Test.

	Relationship Type	Mean Rank	Sum of Ranks	Mann–Whitney *U* Test	Z	*p*-Value
Desire	Married vs. Open monogamous	47.06	2353.00	472.00	−2.99	0.003 *
		31.23	968.00			
	Married vs. Exclusive monogamous	159.93	7996.50	3678.50	−3.25	0.001 *
		122.19	25,414.50			
	Married vs. Open polygamous	35.84	1792.00	233.00	−2.99	0.025
		23.53	353.00			
	Exclusive monogamous vs. Open monogamous	121.27	25,223.50	2960.50	−0.74	0.455
		111.50	3456.50			
	Exclusive monogamous vs. Open polygamous	113.18	23,541.00	1315.00	−1.03	0.303
		95.67	1435.00			
	Open monogamous vs. Open polygamous	24.19	750.00	211.00	−0.51	0.609
		22.07	331.00			

* Statistical significance set at *p* < 0.0083 after Bonferroni corrections.

## Data Availability

The raw data supporting the conclusions of this article will be made available by the authors on request.

## References

[1] Bowlby J. (2012). A Secure Base.

[2] Birnbaum G.E., Reis H.T., Mikulincer M., Gillath O., Orpaz A. (2006). When Sex Is More than Just Sex: Attachment Orientations, Sexual Experience, and Relationship Quality. J. Personal. Soc. Psychol..

[3] Mikulincer M., Shaver P.R. (2003). The Attachment Behavioral System in Adulthood: Activation, Psychodynamics, and Interpersonal Processes. Advances in Experimental Social Psychology.

[4] Mikulincer M., Shaver P.R. (2016). Attachment in Adulthood: Structure, Dynamics, and Change.

[5] Péloquin K., Brassard A., Lafontaine M.-F., Shaver P.R. (2014). Sexuality Examined Through the Lens of Attachment Theory: Attachment, Caregiving, and Sexual Satisfaction. J. Sex Res..

[6] Goldsmith K.M., Dunkley C.R., Dang S.S., Gorzalka B.B. (2016). Sexuality and Romantic Relationships: Investigating the Relation between Attachment Style and Sexual Satisfaction. Sex. Relatsh. Ther..

[7] Stefanou C., McCabe M.P. (2012). Adult Attachment and Sexual Functioning: A Review of Past Research. J. Sex. Med..

[8] Hazan C., Shaver P. (1987). Romantic Love Conceptualized as an Attachment Process. J. Personal. Soc. Psychol..

[9] Valdez C.M., Leonhardt N.D., Busby D.M. (2021). Sexual Passion and Attachment: Sexual Passion Style as a Mediator between Attachment Insecurity and Sexual Satisfaction in Committed Relationships. J. Marital. Fam. Ther..

[10] Birnbaum G.E., Reis H.T. (2019). Evolved to Be Connected: The Dynamics of Attachment and Sex over the Course of Romantic Relationships. Curr. Opin. Psychol..

[11] Florsheim P. (2003). Adolescent Romantic Relations and Sexual Behavior: Theory, Research, and Practical Implications.

[12] Birnbaum G.E. (2015). Like a Horse and Carriage?: The Dynamic Interplay of Attachment and Sexuality During Relationship Development. Eur. Psychol..

[13] Brassard A., Lussier Y., Shaver P.R. (2009). Attachment, Perceived Conflict, and Couple Satisfaction: Test of a Mediational Dyadic Model. Fam. Relat..

[14] Birnbaum G.E., Mikulincer M., Austerlitz M. (2013). A Fiery Conflict: Attachment Orientations and the Effects of Relational Conflict on Sexual Motivation. Pers. Relatsh..

[15] Davis D., Shaver P.R., Vernon M.L. (2004). Attachment Style and Subjective Motivations for Sex. Pers. Soc. Psychol. Bull..

[16] Davis D., Shaver P.R., Widaman K.F., Vernon M.L., Follette W.C., Beitz K. (2006). “I Can’t Get No Satisfaction”: Insecure Attachment, Inhibited Sexual Communication, and Sexual Dissatisfaction. Pers. Relatsh..

[17] Birnbaum G.E. (2007). Attachment Orientations, Sexual Functioning, and Relationship Satisfaction in a Community Sample of Women. J. Soc. Pers. Relatsh..

[18] Gewirtz-Meydan A., Finzi-Dottan R. (2018). Sexual Satisfaction Among Couples: The Role of Attachment Orientation and Sexual Motives. J. Sex Res..

[19] Paul E.L., McManus B., Hayes A. (2000). “Hookups”: Characteristics and Correlates of College Students’ Spontaneous and Anonymous Sexual Experiences. J. Sex Res..

[20] Schachner D.A., Shaver P.R. (2004). Attachment Dimensions and Sexual Motives. Pers. Relatsh..

[21] Mulhall J., King R., Glina S., Hvidsten K. (2008). Importance of and Satisfaction with Sex Among Men and Women Worldwide: Results of the Global Better Sex Survey. J. Sex. Med..

[22] Bogaert A.F., Sadava S. (2002). Adult Attachment and Sexual Behavior. Pers. Relatsh..

[23] Brassard A., Shaver P.R., Lussier Y. (2007). Attachment, Sexual Experience, and Sexual Pressure in Romantic Relationships: A Dyadic Approach. Pers. Relatsh..

[24] Allsop D.B., Leavitt C.E., Saxey M.T., Timmons J.E., Carroll J.S. (2021). Applying the Developmental Model of Marital Competence to Sexual Satisfaction: Associations between Conflict Resolution Quality, Forgiveness, Attachment, and Sexual Satisfaction. J. Soc. Pers. Relatsh..

[25] Bennett-Brown M., Denes A. (2023). Testing the Communication During Sexual Activity Model: An Examination of the Associations among Personality Characteristics, Sexual Communication, and Sexual and Relationship Satisfaction. Commun. Res..

[26] Busby D.M., Hanna-Walker V., Yorgason J.B. (2020). A Closer Look at Attachment, Sexuality, and Couple Relationships. J. Soc. Pers. Relatsh..

[27] Komlenac N., Hochleitner M. (2020). Attachment-Related Anxiety Is Associated with Poor Genital Satisfaction and Sexual Problems in Women. BMC Women’s Health.

[28] Cohen D.L., Belsky J. (2008). Avoidant Romantic Attachment and Female Orgasm: Testing an Emotion-Regulation Hypothesis. Attach. Hum. Dev..

[29] Stephenson K.R., Meston C.M. (2010). When Are Sexual Difficulties Distressing for Women? The Selective Protective Value of Intimate Relationships. J. Sex. Med..

[30] Birnbaum G.E. (2010). Bound to Interact: The Divergent Goals and Complex Interplay of Attachment and Sex within Romantic Relationships. J. Soc. Pers. Relatsh..

[31] McNulty J.K., Wenner C.A., Fisher T.D. (2016). Longitudinal Associations Among Relationship Satisfaction, Sexual Satisfaction, and Frequency of Sex in Early Marriage. Arch. Sex. Behav..

[32] Roels R., Janssen E. (2022). Attachment Orientations, Sexual Behavior, and Relationship Satisfaction in Young, Mixed-Sex Couples: A Dyadic Approach. J. Sex Marital. Ther..

[33] Schoenfeld E.A., Loving T.J., Pope M.T., Huston T.L., Štulhofer A. (2017). Does Sex Really Matter? Examining the Connections Between Spouses’ Nonsexual Behaviors, Sexual Frequency, Sexual Satisfaction, and Marital Satisfaction. Arch. Sex. Behav..

[34] Berli C., Schwaninger P., Scholz U. (2021). “We Feel Good”: Daily Support Provision, Health Behavior, and Well-Being in Romantic Couples. Front. Psychol..

[35] Kaestle C.E., Evans L.M. (2018). Implications of No Recent Sexual Activity, Casual Sex, or Exclusive Sex for College Women’s Sexual Well-Being Depend on Sexual Attitudes. J. Am. Coll. Health.

[36] Stronge S., Overall N.C., Sibley C.G. (2019). Gender Differences in the Associations between Relationship Status, Social Support, and Wellbeing. J. Fam. Psychol..

[37] Ševčíková A., Gottfried J., Blinka L. (2021). Associations among Sexual Activity, Relationship Types, and Health in Mid and Later Life. Arch. Sex. Behav..

[38] Castro Á., Correa A.B. (2023). Psychological and Psychosexual Adjustment in University Students as a Function of Sexual Activity and Relationship Type. Int. J. Sex. Health.

[39] Hehman J.A., Salmon C.A. (2020). Beyond Sex Differences: Predictors of Negative Emotions Following Casual Sex. Evol. Psychol. Sci..

[40] Woerner J., Abbey A. (2017). Positive Feelings After Casual Sex: The Role of Gender and Traditional Gender-Role Beliefs. J. Sex Res..

[41] Sotiropoulou P., Ferenidou F., Owens D., Kokka I., Minopoulou E., Koumantanou E., Pavlopoulou I., Apotsos P., Karvouni M., Koumantarou E. (2021). The Impact of Social Distancing Measures Due to COVID-19 Pandemic on Sexual Function and Relationship Quality of Couples in Greece. Sex. Med..

[42] Mourikis I., Antoniou M., Matsouka E., Vousoura E., Tzavara C., Ekizoglou C., Papadimitriou G.N., Vaidakis N., Zervas I.M. (2015). Anxiety and Depression among Greek Men with Primary Erectile Dysfunction and Premature Ejaculation. Ann. Gen. Psychiatry.

[43] Faul F., Erdfelder E., Buchner A., Lang A.-G. (2009). Statistical Power Analyses Using G*Power 3.1: Tests for Correlation and Regression Analyses. Behav. Res. Methods.

[44] Dennerstein L., Dudley E., Burger H. (2001). Are Changes in Sexual Functioning during Midlife Due to Aging or Menopause?. Fertil. Steril..

[45] Scavello I., Maseroli E., Di Stasi V., Vignozzi L. (2019). Sexual Health in Menopause. Medicina.

[46] Bostani Khalesi Z., Jafarzadeh-Kenarsari F., Donyaei Mobarrez Y., Abedinzade M. (2020). The Impact of Menopause on Sexual Function in Women and Their Spouses. Afr. Health Sci..

[47] Waldinger M.D. (2015). Psychiatric Disorders and Sexual Dysfunction. Handbook of Clinical Neurology.

[48] Banaei M., Azizi M., Moridi A., Dashti S., Yabandeh A.P., Roozbeh N. (2019). Sexual Dysfunction and Related Factors in Pregnancy and Postpartum: A Systematic Review and Meta-Analysis Protocol. Syst. Rev..

[49] Rosen C., Brown J., Heiman S., Leib R. (2000). The Female Sexual Function Index (FSFI): A Multidimensional Self-Report Instrument for the Assessment of Female Sexual Function. J. Sex Marital. Ther..

[50] Zachariou A., Filiponi M., Kirana P.S. (2017). Translation and Validation of the Greek Version of the Female Sexual Function Index Questionnaire. Int. J. Impot. Res..

[51] Hendrick S.S. (1988). A Generic Measure of Relationship Satisfaction. J. Marriage Fam..

[52] Simou P., Moraitou D. (2018). The Association between Mindfulness and Romantic Relationship Satisfaction in Adults. Hell. J. Psychol..

[53] Fraley R.C., Heffernan M.E., Vicary A.M., Brumbaugh C.C. (2011). The experiences in close relationships—Relationship Structures Questionnaire: A method for assessing attachment orientations across relationships. Psychological Assessment..

[54] Tsagarakis M., Kafetsios K., Stalikas A. (2007). Reliability and Validity of the Greek Version of the Revised Experiences in Close Relationships Measure of Adult Attachment. Eur. J. Psychol. Assess..

[55] Bartholomew K., Horowitz L.M. (1991). Attachment Styles among Young Adults: A Test of a Four-Category Model. J. Personal. Soc. Psychol..

[56] Dunkley C.R., Dang S.S., Chang S.C.H., Gorzalka B.B. (2016). Sexual Functioning in Young Women and Men: Role of Attachment Orientation. J. Sex Marital. Ther..

[57] Sagone E., Commodari E., Indiana M.L., La Rosa V.L. (2023). Exploring the Association between Attachment Style, Psychological Well-Being, and Relationship Status in Young Adults and Adults—A Cross-Sectional Study. Eur. J. Investig. Health Psychol. Educ..

[58] Weber R., Eggenberger L., Stosch C., Walther A. (2022). Gender Differences in Attachment Anxiety and Avoidance and Their Association with Psychotherapy Use—Examining Students from a German University. Behav. Sci..

[59] Overall N.C., Cross E.J., Agnew C.R., Harman J.J. (2019). Attachment Insecurity and the Regulation of Power and Dependence in Intimate Relationships. Power in Close Relationships.

[60] Impett E.A., Gordon A.M., Strachman A. (2008). Attachment and Daily Sexual Goals: A Study of Dating Couples. Pers. Relatsh..

[61] Rusbult C.E. (1980). Commitment and Satisfaction in Romantic Associations: A Test of the Investment Model. J. Exp. Soc. Psychol..

[62] Thibaut J.W., Kelley H.H. (2017). The Social Psychology of Groups.

[63] Klusmann D. (2002). Sexual Motivation and the Duration of Partnership. Arch. Sex. Behav..

[64] Sprecher S. (2002). Sexual Satisfaction in Premarital Relationships: Associations with Satisfaction, Love, Commitment, and Stability. J. Sex Res..

[65] Fugère M.A., Chabot C., Doucette K., Cousins A.J. (2017). The Importance of Physical Attractiveness to the Mate Choices of Women and Their Mothers. Evol. Psychol. Sci..

[66] Poulsen F.O., Holman T.B., Busby D.M., Carroll J.S. (2013). Physical Attraction, Attachment Styles, and Dating Development. J. Soc. Pers. Relatsh..

[67] Birnbaum G., Cassidy J., Shaver P. (2016). Attachment and Sexual Mating: The Joint Operation of Separate Motivational Systems. Handbook of Attachment: Theory, Research, and Clinical Applications.

[68] Basson R. (2001). Human Sex-Response Cycles. J. Sex Marital. Ther..

[69] Thomae M., Houston D.M. (2016). The Impact of Gender Ideologies on Men’s and Women’s Desire for a Traditional or Non-Traditional Partner. Personal. Individ. Differ..

[70] Abbott K., Weckesser A., Egan H. (2021). ‘Everyone Knows Someone in an Unhealthy Relationship’: Young People’s Talk about Intimate Heterosexual Relationships in England. Sex Educ..

[71] Ogolsky B.G., Monk J.K., Rice T.M., Theisen J.C., Maniotes C.R. (2017). Relationship Maintenance: A Review of Research on Romantic Relationships. J. Fam. Theo Revie.

[72] Sprecher S. (2001). Equity and Social Exchange in Dating Couples: Associations With Satisfaction, Commitment, and Stability. J. Marriage Fam..

[73] Basson R. (2002). Women’s Sexual Desire—Disordered or Misunderstood?. J. Sex Marital. Ther..

[74] Dienberg M.-F., Oschatz T., Piemonte J.L., Klein V. (2023). Women’s Orgasm and Its Relationship with Sexual Satisfaction and Well-Being. Curr. Sex. Health Rep..

[75] Lawrance K., Byers E.S. (1995). Sexual Satisfaction in Long-term Heterosexual Relationships: The Interpersonal Exchange Model of Sexual Satisfaction. Pers. Relatsh..

[76] Józefacka N.M., Szpakiewicz E., Lech D., Guzowski K., Kania G. (2023). What Matters in a Relationship—Age, Sexual Satisfaction, Relationship Length, and Interpersonal Closeness as Predictors of Relationship Satisfaction in Young Adults. Int. J. Environ. Res. Public Health.

[77] Park H.G., Leonhardt N.D., Johnson M.D., Muise A., Busby D.M., Hanna-Walker V.R., Yorgason J.B., Holmes E.K., Impett E.A. (2023). Sexual Satisfaction Predicts Future Changes in Relationship Satisfaction and Sexual Frequency: New Insights From Within-Person Associations Over Time. Personal. Sci..

[78] Fallis E.E., Rehman U.S., Woody E.Z., Purdon C. (2016). The Longitudinal Association of Relationship Satisfaction and Sexual Satisfaction in Long-Term Relationships. J. Fam. Psychol..

[79] Sims K.E., Meana M. (2010). Why Did Passion Wane? A Qualitative Study of Married Women’s Attributions for Declines in Sexual Desire. J. Sex Marital. Ther..

[80] Muise A., Harasymchuk C., Day L.C., Bacev-Giles C., Gere J., Impett E.A. (2019). Broadening Your Horizons: Self-Expanding Activities Promote Desire and Satisfaction in Established Romantic Relationships. J. Personal. Soc. Psychol..

[81] Birnbaum G.E., Reis H.T., Mizrahi M., Kanat-Maymon Y., Sass O., Granovski-Milner C. (2016). Intimately Connected: The Importance of Partner Responsiveness for Experiencing Sexual Desire. J. Personal. Soc. Psychol..

[82] Casado-Espada N.M., De Alarcón R., De La Iglesia-Larrad J.I., Bote-Bonaechea B., Montejo Á.L. (2019). Hormonal Contraceptives, Female Sexual Dysfunction, and Managing Strategies: A Review. J. Clin. Med..

[83] Fraley R.C., Hudson N.W., Heffernan M.E., Segal N. (2015). Are Adult Attachment Styles Categorical or Dimensional? A Taxometric Analysis of General and Relationship-Specific Attachment Orientations. J. Personal. Soc. Psychol..

[84] Latkin C.A., Edwards C., Davey-Rothwell M.A., Tobin K.E. (2017). The Relationship between Social Desirability Bias and Self-Reports of Health, Substance Use, and Social Network Factors among Urban Substance Users in Baltimore, Maryland. Addict. Behav..

[85] Kirchherr J., Charles K. (2018). Enhancing the Sample Diversity of Snowball Samples: Recommendations from a Research Project on Anti-Dam Movements in Southeast Asia. PLoS ONE.

